# Chronic Disease Management in a Nurse Practitioner-Led Clinic: An Interpretive Description Study

**DOI:** 10.1177/23779608241299292

**Published:** 2024-12-13

**Authors:** Natalie Floriancic, Anna Garnett, Lorie Donelle

**Affiliations:** 1 6221 Western University, London, Canada; 2 Western School of Nursing, London, Canada; 3 College of Nursing, 2629 University of South Carolina, London, SC, USA

**Keywords:** nurse practitioner, advanced practice nurse, chronic disease, interprofessional, primary healthcare

## Abstract

**Introduction:**

Nurse practitioner-led clinics (NPLC) represent a model of care that has the potential to enhance primary healthcare delivery to community-dwelling adults who are living with chronic disease by providing greater access and continuity of care and reducing the burden on acute care settings. However, there is limited understanding of nurse practitioners’ experiences and perspectives on supporting adults in chronic disease management within an NPLC model of care. Increased understanding would contribute to our ability to evaluate the effectiveness of the NPLC model of care for chronic disease management.

**Objective:**

This study explored current chronic disease management practices implemented by nurse practitioners within NPLC throughout the Province of Ontario.

**Methods:**

A qualitative study was implemented using interpretive description. In-depth interviews were conducted between January 2021 and May 2021 with nurse practitioners who were practicing within NPLCs across Ontario. Data was analyzed using thematic analysis.

**Results:**

Eleven interviews were completed with nurse practitioners who provided care to community-dwelling adults who were managing chronic disease in a range of geographic settings. Resultant themes included: the nurse practitioner role in bridging access to patients who fall between the cracks, benefits of interprofessional care, meeting a patient where they are at, and addressing healthcare system burden.

**Conclusion:**

Results suggest that NPLCs are well-positioned to support community-based patients living with chronic disease through provision of on-site interprofessional care, continuity in service provision and increased access to primary healthcare services. This article provides insights into the nurse practitioner led primary healthcare model and how it can facilitate access to services, foster patient self-management and provide a successful alternative model of care.

## Introduction

A strong primary healthcare system is recognized as the cornerstone of health systems and leads to better health outcomes, improved patient experiences and lower healthcare costs (Haj-Ali et al., 2021). The leading cause of preventable death and disability and increased healthcare costs worldwide is chronic disease (Heale et al., 2018a; Lukewich et al., 2014; Russell et al., 2009). Primary healthcare providers play an important role in the delivery of ongoing community-based chronic disease management (Heale et al., 2018; Poghosyan et al., 2017). Similarly, primary healthcare organizations are well situated to oversee the management of chronic conditions through the provision of continuity in care, care coordination, and comprehensive service delivery (Black et al., 2020; Reynolds et al., 2018). Until recently, primary healthcare in Canada was largely delivered by physicians working as independent practitioners or small physician groups with a focus on basic medical services (Marchildon & Hutchison, 2016). Over the past 10 years Canada has made advancements in primary healthcare reform aimed at increasing access and improving the quality of primary healthcare services available to its citizens (Black et al., 2020; Côte et al., 2019; Heale et al., 2018; Keith & Askin, 2008; Marchildon & Hutchison, 2016; O’Rourke & Higuchi, 2016).

There are currently three models of care operating in the province of Ontario including family health teams (FHT), community health centers (CHC), and nurse practitioner-led clinics (NPLC). The main difference between each model of care is the role of the physicians and nurse practitioners (NPs). FHTs are physician-led organizations that support an interprofessional team approach with physicians, NPs, registered nurses, social workers, dietitians, and other allied health professionals as part of the team (Heale et al., 2018a). Within this model of care patients are directly registered to individual physicians (Heale et al., 2018a). CHCs deliver health, social and community services through an interprofessional team structure as well (Dahrouge et al., 2014). This model of care is physician-led; however, it differs from FHTs as physicians and NPs both have patients directly registered to them individually with NPs having lower acuity patients registered to them (Dahrouge et al., 2014). The NPLC model of care provides primary healthcare services to clients with physicians employed as consultants (DiCenso et al., 2010; Heale et al., 2018). This model of care enables NPs to optimize their full scope of practice and fosters an equity-oriented approach within primary healthcare. Chronic disease management led by NPs in autonomous practices such as NPLCs has received limited evaluation.

## Review of Literature

The title of NP was legislated in the province of Ontario in August 2007 (Mian et al., 2012). The role of the primary healthcare NP in Canada focuses on episodic illness, health promotion, disease prevention, and chronic disease management (Mian et al., 2012; O’Rourke & Higuchi, 2016). The current scope of practice for NPs in Ontario is the broadest of all Canadian provinces and enables NPs to assess and communicate diagnoses, order diagnostic testing, complete specialist referrals, and prescribe medications including narcotic and controlled substances when indicated (College of Nurses of Ontario, 2021).

High healthcare costs, poor quality of life, inappropriate use of emergency departments and urgent care facilities, functional decline, and disability are all associated with living with multiple chronic conditions (Lukewich et al., 2018; Makovski et al., 2019). When individuals with multiple chronic conditions and medical complexity experience challenges accessing primary healthcare services, the NP role is well-situated to help reduce health inequities experienced by this population (Heale et al., 2018b).

The chronic care model was developed as a way of improving care for clients with chronic diseases in a range of healthcare settings (Dunn & Conard, 2018). This model has been used to examine primary healthcare impacts of key biometrics such as glycated hemoglobin, blood pressure, body mass index and cholesterol as well as the delivery of community-based programs such as diabetes primary prevention through the Young Men's Christian Association (Dunn & Conard, 2018). The chronic care model framework looks at the impact of the community, self-management supports, delivery system designs, decision supports, and clinical information systems on chronic disease care (Pan American Health Organization, 2021). This study used this framework to analyze the organizational approach to caring for clients with chronic disease within the NPLC setting.

There is limited knowledge regarding how chronic disease management services are provided within NPLCs. The purpose of this study was to explore the NP experience in providing chronic disease management within the NPLC model of care in the Province of Ontario to gain a better understanding of their perception of this care model to meet the needs of community-based adults living with chronic disease. The research question guiding this investigation is: What is the experience of NPs in providing comprehensive chronic disease management care to patients in the context of NPLCs in Ontario?

## Methods

### Design

This study used Thorne's (2004) interpretive description methodology, an approach that is intended to answer research questions about health and illness experiences from holistic, interpretive, and relational perspectives (Burdine et al., 2020; Thorne et al., 2004). With a philosophical alignment with interpretive naturalistic orientations which acknowledges the contextual nature of human experience interpretive description allows for the existence of shared realities (Thorne et al., 2004). This approach supports NP participants in this study as “situated knowers” who can contribute to solving nursing problems and fulfill their social mandate to promote health and wellbeing (Reed, 2020; Thorne, 2016).

### Research Question

What is the experience of NPs in providing comprehensive chronic disease management care to patients in the context of NPLCs in Ontario?

### Sample

A purposive sample was used to obtain in-depth descriptions of NPs experience providing primary care services to patients with chronic diseases. Members of the research team used their knowledge of NPLC operations to identify clinics who could provide potential study participants. Eligible NPLC organizations were approached through publicly accessible administrative emails. Eighteen organizations in total were approached through email for participation, 11 responses were received and no participants dropped out of the study. Inclusion criteria for participation in this study included NPs currently practicing within an NPLC in Ontario for a minimum of 12 months who were able to speak and read English and be willing to discuss their experience providing primary care services to patients diagnosed with chronic diseases. Exclusion criteria included NPs not currently practicing within an NPLC. Ethical approval for this study was obtained from the research ethics board.

An appropriate sample of participants is, in part, a reflection of the existing background literature that a phenomenon occurs within clinical practice and an in-depth exploration of the subjective nature is required; in this circumstance a small number of individuals will produce findings that are worth noting (Thorne, 2016). The sample size for this study was in part driven by other qualitative works using interpretive description who reported sample sizes ranging from 10 to 15 participants (Northwood et al., 2021; Thorne et al., 2004; Yous et al., 2019). Data collection and analysis were completed iteratively, and the research team continued recruiting participants until data saturation was achieved.

### Statistical Analysis

The primary data collection was completed using telephone interviews because the eleven study participants were located across several geographic regions throughout the province of Ontario. These in-depth interviews were completed by the first author with supervision and advisement from the second author supported by a semistructured interview guide and through the addition of ad hoc questions as appropriate (Appendix A). The chronic care model was used to inform interview guide development due to its relevance as an organizational approach to informing care of patients with chronic disease in primary healthcare settings (Improving Chronic Illness Care, 2021). The first two participant interviews were reviewed with the research team to ensure the interview guide was eliciting data that would meet the study objectives. Adjustments to the interview guide were made and additional questions were added in subsequent interviews in accord with the direction and flow of the interviewee responses.

Each interview lasted 45 to 60 minutes and were digitally audio-recorded and uploaded to a secure computer for data analysis. Data was uploaded to NVivo 12 qualitative software and conceptual themes were derived inductively (Nowell et al., 2017). Analysis was an iterative process which involved frequent communication with the full research team. Direct discussion with study participants for review of transcripts was not feasible for this study due to time constraints. Initial coding was completed through analysis of each transcript with highlighting and constant comparison of key words, phrases, and concept development (Burdine et al., 2020; Nowell et al., 2017). The initial codes were then grouped into common categories and subcategories. Confirmability was achieved by including direct quotes from study participants to demonstrate each theme and link them to individual experiences. The final developed themes offered insight into the organizational approach to providing primary healthcare services to clients with chronic disease within NPLCs (Pan American Health Organization, 2021; Nowell et al., 2017).

Interpretive description studies borrow principles of constant comparative analysis to support interpretations from the analysis that are consistent and plausible (Burdine et al., 2020). Researchers took several steps to ensure qualitative rigor or trustworthiness was maintained throughout the study process (Lincoln & Guba, 1985; Nowell et al., 2017). A consistent approach was used for data collection and analysis including using a semistructured interview guide, maintaining reflexive notes following each interview, and through the use of field notes where discussions with the research team and other information such as the context and tone of interviews were documented (Phillippi & Lauderdale, 2018). Analyst triangulation was used throughout the data analysis process to test credibility with review of completed transcripts, initial coding, and iterative comparison of findings among the research team (Lincoln & Guba, 1985; Nowell et al., 2017). Dependability and transferability were addressed by the research team through collection of an audit trail which included electronically recorded methodological decisions and rationale through study design, data collection, and analysis (Lincoln & Guba, 1985; Nowell et al., 2017). Field notes were also completed throughout the study process to record personal reflections of values, interests, and insights from the research team.

According to Thorne (2016) a sound critique of qualitative research beyond the surface level also includes consideration of moral defensibility, disciplinary relevance, and pragmatic obligation. Moral defensibility refers to the requirement of applied science researchers to provide rationale that will link findings to a potential benefit for those we serve (Thorne, 2016). Moral defensibility was considered by researchers in this study design as they aimed for study findings to be used to inform primary healthcare practice and primary healthcare system reform (Thorne, 2016). Disciplinary relevance addresses the issue of whether the research findings will contribute to the development of the disciplinary science (Thorne, 2016). The nursing discipline has the potential to benefit from the knowledge produced in this study through clinical practice and policy development highlighting disciplinary relevance (Thorne, 2016). Pragmatic obligation requires researchers to acknowledge that a discipline may apply findings in practice prior to them being scientifically proven due to the coexisting realities present in practical sciences (Thorne, 2016). Pragmatic obligation was considered by the research team as this study explored current NP practice already implemented in several NPLCs with the understanding that results may impact future clinical practice considerations (Thorne, 2016).

## Results

Data was collected from January 2021 to May 2021. Eleven NPs participated in this study with ages ranging from 31 to 61 years of age or older. Nine participants identified as female, and two participants identified as male. Participants were practicing in eight different NPLC locations out of currently 25 operating in Ontario. Years of experience among participants ranged from 3 years to more than 16 years.

Participants described the impacts of the social determinants of health on their ability to support adults’ chronic disease management. Participants identified financial constraints, limited health literacy, food insecurity, homelessness, complex trauma history and insufficient health resource allocation as obstacles that hindered health supporting behaviors. Four main themes were identified within the data including: (1) bridging access to clients who fall between the cracks, (2) benefits of interprofessional care, (3) underappreciation of NP knowledge and skill level, and (4) addressing healthcare system burden.

### Theme 1: Bridging Access to Clients Who Fall Between the Cracks

Study participants frequently discussed how the social determinants of health negatively impacted their client's health and wellbeing. Several factors that may impact an individual's health status included education, income, housing, food security, language, culture, gender, sexual orientation, and healthcare system constraints including funding and limited provider availability. NP participants reported that many of their clients experienced homelessness and impoverishment. NPs also described the challenge of providing care to clients who have previously used walk-in clinics and emergency departments for chronic disease management. A study participant stated:

We have a lot of patients with low education, low understanding of the healthcare system…low education turns into low housing, to low income to then poor health and as you know just kind of trickles right down. (NP04)

NPs spoke about the need to understand each client's social circumstances to determine its potential impact on their chronic disease. Clients without access to adequate housing or food required additional supports to provide access to basic needs in addition to the medical support they required. Study participants discussed their challenges in gaining access to resources for clients to support their health. NPs described limited options for coverage of medications, rehabilitative services, dental care, and counseling services for clients. NPs demonstrated their resourcefulness by working with community partners to maximize available resources and funding to better support client needs. For example, study participants discussed partnering with Canadian Mental Health Association locations for counseling and mental health services, local refugee centers to support newcomers’ access to primary healthcare and translation services, community food banks, and local rapid access clinics to support clients with substance use disorders. A study participant stated:

We do have a lot of clienteles that we call the working poor because they may not have benefits with their job or may not have sick benefits, may not have drug plans. Or we have a lot of people on Ontario Works and Ontario Disability Support Program so some medications will be covered but not all of them. So, these social determinants there's lots of risk of homelessness, people in precarious living situations where they can’t afford all their bills and rent so they’ll pay rent but then have food insecurity. (NP07)

NP participants discussed the importance of their role in advocating for systemic change to address the ongoing disparity their clients’ experience. Local, provincial, and federal governments have been lobbied by NPs and their associations regarding the need for additional supports in housing, access to food, supports for newcomers facing deportation, medication coverage, and supports for clients living with disabilities. As the primary healthcare provider this advocacy role was taken on by NPs to promote client-centered care. A study participant stated:

Often people don’t have homes, they don’t have regular lifestyles or money to afford good food. It's hard for them to get to appointments so you need to be flexible in terms of somebody walks in with a need you gotta be prepared to squeeze them in or address it. Making access to resources shapes the chronic disease management here. (NP05)

NPs worked to increase access to resources for their clients through use of compassionate care programs and partnerships with pharmaceutical companies who were willing to provide medication samples to those without the ability to afford them. Local members of the provincial parliament were regularly contacted regarding the need for increased social supports and NPs also invited them to visit their clinic sites to demonstrate the gaps in resource access.

### Theme 2: Benefits of Interprofessional Care

Multimorbidity and chronic disease such as diabetes, chronic obstructive pulmonary disease (COPD), hypertension, coronary artery disease, mental health conditions and substance use disorders are common complex presentations seen within NPLCs. An interprofessional team approach to provide comprehensive care to clients with complex presentations include collaboration among NPs, consulting physicians, registered nurses, community health workers, social workers, pharmacists, and all members of the healthcare team.

There is recognition and understanding within these teams that care can be optimized and efficient through collaboration when managing older adults who present with increasingly complex health conditions. A study participant stated:

Having a team is really beneficial because as the population ages and we’re seeing more and more chronic disease I think having each person in that team work to their scope is really important. Because it makes for a much more efficient system ensuring that patients are getting seen and ensuring that they are being managed properly and it's a really great use of each provider's time. (NP02)

Within the interprofessional team study participants discussed their perspective on role hierarchy and development of client care plans. Unlike traditional models of care NPs felt all members of the healthcare team were comfortable approaching them to collaborate when developing client care plans. Clients with complex chronic disease often required medical and social issues be addressed during clinic appointments. One study participant stated:

In an NPLC with the full team the hierarchy is not there as much as in a traditional physician based practice where whatever the physician says is what goes. Whereas in the NPLC if any of the allied team members give me a suggestion, I don’t feel that we have that hierarchy where they can’t come to us with that or change the plan because they have different or better ideas. (NP04)

Study participants also discussed the benefits of working with NP colleagues with unique skill sets that contribute to holistic care. Participants felt the NPLC model supports continuous collaboration between providers and due to the education and training requirements of NPs they often bring a variety of experiences to their practice. A study participant stated:

With nurse practitioners we all come to each other for certain things. Everybody has their strengths and their interest in their own practice we have some nurse practitioners that have more of a cardiac background, some that have more of a home care background. I’, mental health and other things, so we really just kind of support each other and ask questions and bounding back and forth it's pretty symbiotic really. (NP03)

Within the NPLC model of care a consulting physician is a part of the healthcare team and is available to practitioners for consultation if required. NP providers can reach out to consulting physicians for specific clients for care plan discussions based on their discretion. All 11 NP participants expressed gratitude and described the benefits of having a consulting physician as a part of the interprofessional team. A study participant stated:

We do have a great collaborative physician we would be able to go to the collaborative physician and have a conversation so our patients wouldn’t have any less access to care because they see a nurse practitioner. (NP02)

Collaborative practice allows for comprehensive care to be provided within the constraints of time and financial resources allocated to primary healthcare organizations. As described by participants, registered practical nurses, registered nurses, dietitians, pharmacists, physiotherapists, social workers, and community health workers all contribute to important aspects of chronic disease management, monitoring, and support continuity of care for clients.

### Theme 3: Underappreciation of Nurse Practitioner Knowledge and Skill Level

The NPLC model of care has existed in Ontario since 2007 yet study participants spoke about the lack of awareness of the NP role within primary healthcare among clients in their practice and other practitioners in the healthcare community. Study participants discussed how clients often misunderstood the scope of practice of NP-led care believing there were limitations in their ability to order tests, prescribe medications or make specialist referrals. Several participants discussed their experience in explaining the purpose of the NPLC model of care to clients to ensure they understood their role and the resources they would have access to through the interprofessional team with one participant sharing, “not everybody knows what a nurse practitioner does I think they are shocked when they find out exactly what we do” (NP03). Misperceptions about the NP role were also discussed as a study participant stated:

A lot of people think when they’re coming to a nurse practitioner led clinic that we’re a temporary stop to hold them over until they find a doctor…I think we still have a long way to go to promote our profession in the public eye. (NP04)

There were several accounts from study participants about the complexity of clients in their care and the NPs’ ability to provide comprehensive management for chronic disease presentations. A study participant stated:

I think one thing that is underappreciated is the depth of knowledge and the high level of skill the nurse practitioners in this setting need to have. I don’t think we in the NPLCs acknowledge it and I think people should be given credit for the just fabulous work that they do with a very challenging population on the whole. Some NPLCs are seeing relatively healthy people but the majority are not. (NP05)

Similarly, another participant discussed the lack of recognition from the medical community and government sectors related to the complicated health presentations they regularly manage in their practice stating, “it is pretty common knowledge that we take clients that other models [clinicians] reject because of their complexity so I think there's some recognition of it, but not great recognition” (NP07). Study participants discussed their role and scope of practice in relation to providing chronic disease services to their clients. Several participants discussed how the NP role within primary healthcare is ideally suited for chronic disease management. A study participant stated:

The nurse practitioner in my humble opinion is ideally positioned to provide chronic disease health services based on common elements of their vision, mission and professional values…nurse practitioners approach their scope of practice and their role with the intent to utilize their role and their personal attributes to support the provision of innovative and high-quality care for their clients. (NP09)

Study participants discussed the need to adapt care plans based on clients’ access to medications, rehabilitation services, and basic needs. Within the interprofessional team NPs acted as leaders in seeking out resources and skills from allied health professionals to help support client needs. For example, clients who were unable to independently attend required appointments in clinic or the community would be assigned caseworkers who become a part of the care team in supporting their health needs or NPs made home visits to ensure primary healthcare remained accessible.

### Theme 4: Addressing Healthcare System Burden

The NPLC model of care is one of the four different primary healthcare models currently implemented in Ontario and the participants sampled in this study reported positive experiences with this model. Study participants discussed the need for increased access to primary healthcare services to reduce the use of emergency departments for nonemergent healthcare needs. For example, “we need more of this type of care [NPLC] where we can spend time with patients so that they don’t end up in emerge [Emergency Department] the patients who didn’t get proper primary healthcare that's why they are there” (NP08). NP participants spoke about clients who did not have access to primary healthcare services for several years and were accessing emergency departments to manage their chronic diseases including hypertension, diabetes, and COPD. Study participants also emphasized the importance of optimizing their scope of practice to help reduce wait times for clients who require specialized healthcare services. A study participant stated:

I think working to your scope of practice is really important because that way you’re able to provide full spectrum care for your patients and limit the amount of specialist referrals that are set therefor reducing the amount of burden on the system, reducing the amount of wait times before the patient is able to get that appointment and make modifications to their care…I would say we manage them at a high level reducing the burden on the system. (NP02)

Study participants discussed the common perception that individuals with complex healthcare needs are considered time consuming and therefore less desirable to physicians practicing within a predominately medical model of care who rely on fee-for-service funding. Several participants stated they often accept clients with complex presentations who are unable to find primary healthcare providers due to their health history and current needs. Within the community clients who had previously applied but were denied care from CHCs, FHTs, and single physician practices, applied to the NPLCs to access primary healthcare services. The NPLC model of care has a mandate to provide primary healthcare services to individuals without a current primary healthcare provider. As a result, the NPLC model of care has the potential to help fill in gaps in access to primary healthcare services. A study participant stated:

Our mandate is we only take people that are not rostered to anybody else. We won’t see client from other providers but also create the opening for people that they have no provider, no doctor, no nurse practitioner and that's important. (NP07)

Throughout this study participants shared the various benefits and challenges associated with the NPLC model of care. Many of the issues were related to systems level constraints such as funding and resource allocation. Each of the identified themes highlighted the potential applications for this model of care to provide effective chronic disease management.

## Discussion

The findings from this qualitative research study enhance our understanding of NPs experience in providing chronic disease management within the NPLC model of care. Four themes emerged from the results including: (1) bridging access to clients who fall between the cracks, (2) benefits of interprofessional care, (3) underappreciation of NP knowledge and skill level, and (4) addressing healthcare system burden.

### Bridging Access to Clients Who Fall Between the Cracks

Key among the findings was the challenges faced by NPs to maximize available resources for their clients. Findings suggest that NPs worked to establish partnerships with community organizations to increase access to primary healthcare services. Their experiences highlight the need for accessible resources that support chronic disease management. NP participants highlighted the constant need to adapt care plans and settle for what is feasible over what is recommended. Our analysis supports Lee et al.’s (2021) findings that publicly funded healthcare provider services without access to coverage for outpatient resources such as medications diminishes effectiveness of the healthcare system. Historically universal access to healthcare within Canada has been met with increasing constraints particularly in access to primary healthcare services (Lee et al., 2021; Martin et al., 2018). As such, the findings highlight the need for more resources and innovative approaches to allow for better management of chronic disease through primary models of care (Lee et al., 2021; Martin et al., 2018).

### Benefits of Interprofessional Care

Implementing interprofessional care was a key component in providing comprehensive chronic disease management to clients. NPs readily acknowledged its benefits noting their expanded scope of practice and clinician autonomy enabled them to effectively collaborate with members of the team from other health professions. This finding is corroborated in the literature and adds emphasis to the need for team-based care to allow providers to share the workload and leverage on another's expertise (Flood et al., 2023; Fowler et al., 2020; Grant et al., 2024; Martin et al., 2018).

Leadership was a key component to successfully implementing interprofessional collaboration. NPs acknowledged their education and training prepared them to take on leadership roles and promote collaborative practice. Study participants discussed their focus on optimizing scope of practice within their teams and eliminating role hierarchy. These findings are corroborated in the literature as the presence of NPs supports successful implementation among interprofessional primary healthcare teams (Fowler et al., 2020; Grant et al., 2024; Martin et al., 2018). A recent scoping review also highlighted the need to rethink how NPs in clinical settings can effectively collaborate to address the growing complexities within the healthcare system (Sirimsi et al., 2022).

Study participants expressed appreciation for the ability to work with consulting physicians as a part of the healthcare team in the NPLC model of care. NPs promoted shared decision making among members of the healthcare team to provide comprehensive chronic disease management to clients. The current study findings extend the literature by increasing our understanding of the NP experience in providing team-based primary care services. Within the literature there is discussion about the positive impacts of shared decision making on client outcomes (Sirimsi et al., 2022).

### Underappreciation of Nurse Practitioner Knowledge and Skill Level

Study participants felt there was still a significant lack of awareness and understanding of the NP role in primary care from their clients and other healthcare professions. Many NPs advocated for increased recognition of the NP role within NPLCs at local government levels. This finding corroborates other perspectives presented in the literature which note that role clarity amongst primary healthcare teams is imperative to optimize team functioning and client outcomes (Kilpatrick et al., 2021). Study findings call attention to the continued role ambiguity within primary healthcare systems and the need for improved NP role clarity. A recent scoping review highlighted the barriers to NP implementation within primary healthcare due to a lack of understanding NP role and scope of practice (Torrens et al., 2020).

Another key finding was the NPs ability to provide competent and safe care to complex clients with chronic conditions. Findings suggest that NPs relied on their training and education to provide holistic care addressing the physical and social needs of their clients. The social determinants of health including income, education, housing, and food security had to be considered when developing client care plans. This finding is corroborated in a recent observational study that noted NPs provide intensive care to the benefit of clients with complex clinical and social needs (Fraze et al., 2020).

### Addressing Healthcare System Burden

Study participants expressed concern over the increased prevalence of chronic disease and resulting healthcare system burden. Our analysis supports Steffler et al.’s (2021) findings that within the province of Ontario much higher prevalence of chronic disease has been reported over the past several years. While NPs recognized the need to provide chronic disease management in alignment with standards of care many were conflicted as they were told care plans were not feasible for their clients. Like Steffler et al. (2021) our study findings determined that increased amounts of health system resources beyond what is currently available are required to provide adequate primary healthcare services to clients (Grant et al., 2024).

To provide adequate chronic disease management services attention must be given to providing resources and funding to NP roles within primary healthcare and the NPLC model of care. Study participants expressed concern over the lack of response received from lobbying local government officials for increased funding and resource allocation. In these instances, the NPs helped increase political involvement amongst advanced practice nurses to promote primary healthcare system reform. This finding is corroborated in the literature where researchers note that NPs have the potential to be definitive change agents at the intersection of practice, policy, and leadership (Rosa et al., 2020).

## Strengths and Limitations

Limitations of this study included sampling of NPs without additional sampling of clients who received care from the NPs as well as other healthcare providers within the NPLCs. This could provide insight into different lived experiences of individuals within this healthcare setting. Another limitation of this study was the inability to complete member checking with study participants due to time constraints. NP participants would have been offered opportunity to add to the study findings and researchers could ensure NP perspectives were adequately represented. Strengths of this study included collecting data from NPLCs located across five separate geographic regions in Ontario. This study also applied use of the chronic care model framework to guide data collection and analysis and the research teams’ approach to ensuring qualitative rigor was maintained throughout the study process.

## Implications for Nursing Practice

The knowledge gained from this study has generated implications for nursing practice. Throughout the literature NP commitment to the practice value of enhancing holistic care is evidenced (Gordon et al., 2019). Study participants discussed their roles in providing comprehensive primary care services safely and effectively to clients with complex chronic disease. Within this study NPs effectively sought out community partners to better provide holistic care to clients. For example, partnerships with local refugee centers allowed them to provide access to primary care services to newly immigrated individuals and in turn they were offered translation services during health-related appointments to promote capacity building and client centered care. Additionally, partnerships with local Canadian Mental Health Association locations and rapid access clinics allowed NPs to work with community organizations in support of patients with mental health and substance use disorders. The findings suggest that providing networking opportunities to community organizations to maximize resources that are already allocated could increase access to primary healthcare services.

Study participants also discussed their approaches in building capacity in colleagues. This model of care represents a potential approach to multidisciplinary collaboration within primary care. Study participants discussed their appreciation for working with multiple different health professionals and spoke to the positive impact this had on client care. NPs are knowledgeable and possess a unique skill set to provide health promotion and chronic disease management services. These findings suggest that primary health care roles designed for NPs to practice to their level of advanced education and practice in collaboration with other health professionals can help decrease health disparities (Peacock & Hernandez, 2020).

## Conclusion

This study sought to examine NP experiences in providing chronic disease management to clients within NPLCs. Complex social needs and healthcare system constraints compounded the challenges faced by NPs providing chronic disease management services to their clients. Interprofessional collaboration among multiple different health professions led to increased access, optimized scope of practice and comprehensive chronic disease service provision. NP autonomy contributed to NPs ability to provide competent, safe, and effective care to clients with chronic conditions. Lastly, NPLCs regularly accepted complex clients who did not have access to primary healthcare providers because of an overwhelmed primary healthcare system. Further research is required to inform primary healthcare system reform and NP role development.
